# The effect of Ebola Virus Disease outbreak on hand washing among secondary school students in Ondo State Nigeria, October, 2014

**DOI:** 10.11694/pamj.supp.2015.22.1.6614

**Published:** 2015-10-11

**Authors:** Olayinka Stephen Ilesanmi, Faith Osaretin Alele

**Affiliations:** 1 Department of Community Health, Federal Medical Centre, Owo, Ondo State, Nigeria

**Keywords:** Ebola, Hand-washing, Student, Infection, Ebola Virus Disease

## Abstract

**Introduction:**

Hand washing with soap and water is one of the cheapest, most effective ways of limiting the spread of Ebola Virus Disease (EVD). Despite its importance the prevalence of hand washing was low before the EVD outbreak in Nigeria. This study aimed at determining the factors associated with improved hand washing practices following the EVD outbreak.

**Methods:**

A descriptive cross sectional study of 440 students from a secondary school in Owo, Ondo State was done. Data was collected in October 2014 when Nigeria was yet to be declared EVD free. Systematic random sampling was used. A semi-structured, interviewer administered questionnaire was used. Data was analysed with epi info version 7, descriptive statistics were done, Chi square test was used for the assessment of significant associations between proportions. Determinants of good hand washing practices was identified using logistics regression analysis at 5% level of significance.

**Results:**

Of 440 respondents, mean age was 13.7±1.9 years. Females were 48.2%. Only 4.6% have never heard of Ebola Virus Disease.Level of hand washing with soap and water improved by62.6%. Significant improvement in hand washing was in 75.8% of those who heard through social media (p < 0.001), 70.5% of Newspaper readers(p < 0.001), 65.6% of radio listeners (p = 0.001), 75.4% of family members p < 0.001, 76.3% talk in church p < 0.001, 77.6% peers p = 0.02, 72.4% TV p < 0.001.Change in hand washing practices was associated with watching television (AOR: 2.2; CI 95%: 1.1-4.3) and listening to health education in church (AOR: 2.4; CI 95%: 1.2-4.7).Major reason for change in hand washing practices was because of EVD deadly nature, 170(40.5%).

**Conclusion:**

Watching health education messages on television and listening to it in church are the determinants of change in hand washing practices. Promotion of hand washing with soap and water needs to be sustained to prevent other diseases. Training of students on prevention of EVD was conducted in selected schools.

## Introduction

The last outbreak of Ebola Virus Disease caught Nigerians unaware, most interventions were not planned. The case fatality rate in Nigeria was 40% as at September, 2014. Nigeria was declared Ebola free in October, 2014 [1, 2]. In July 2014, the Federal Ministry of Health, with guidance from the Nigeria Centre for Disease Control (NCDC), declared an Ebola emergency in Lagos. Lagos has a population of 21 million. It is a regional hub for economic, industrial, and travel activities hence communicable diseases can spread easily [3].

In the current outbreak in West Africa sub-region, the majority of cases have occurred as a result of human-to-human transmission. Infection occurs through direct contact with broken skin, mucous membranes, blood and other body fluids or secretions (stool, urine, saliva, semen) of infected people [4].

Rapid response using all available public health assets was immediately implemented in Nigeria. These include the use of all available social medial to spread information about Ebola Virus Disease (EVD), personal hygiene, good public health practices and safety measures confer some protection [5]. Hand washing received high attention in Nigeria as a result of EVD outbreak.

Washing hands with soap and water is one of the most effective means of preventing infection [6]. Hand washing and personal hygiene has been shown to reduce the rate of transmission. This study aimed at determining the factors associated with improved hand washing practices following the EVD outbreak.The result of this study will be used to improve practices that will help in preventing the spread of future outbreak.

## Methods

The study was conducted in Owo, Ondo State, Nigeria. The study population was one of the secondary schools in Owo. The school was selected by using simple random sampling method out of the secondary schools in Owo, Ondo State. All consenting selected studentswere studied. A descriptive cross sectional design was used

### Sampling methods

Stage 1: from the list of all public secondary schools in Owo metropolis, number was assigned to each of the schools, one was selected by simple random sampling. The school corresponding to the selected number was chosen. Stage 2: from the list of students in each class systematic random sampling was used to select participants until the required sample size was achieved.

### Sample size determination

The sample size was calculated using the Leslie Kish formula for sample size determination for proportion. Minimum desired sample size calculated was 402 using the standard normal deviate of 1.96 which corresponds to 5% level of significance with a prevalence of 50%and 5% non-response rate.

### Data management

A semi-structured interviewer administered questionnaire was used. Data was collected in October 2014 when Nigeria was yet to be declared Ebola Virus Disease free. Questionnaires were checked for omissions and errors after collection and correction were made where necessary. Data was analysed with epi info version 7, descriptive statistics were done, Chi square test was used for the assessment of significant associations between proportions. Determinants of good hand washing practices was identified using logistics regression analysis at 5% level of significance.

### Ethical consideration

Ethical approval was obtained from the Health Research Ethics Committee of Federal Medical Centre, Owo, Ondo State. Participants were made to understand that participation is voluntary and there was no consequences for non-participation. All information obtained was kept confidential.

## Results

The mean age of respondents was 13.7 years ± 1.9 standard deviation. Table 1 shows the sociodemographic characteristics of respondents. Age range from 10-19 years. Only 133(30.23%) were 15 years and above, while females were 212(48.18%). Other sociodemographic characteristics are shown in Table 1.

**Table 1:** Socio-demographic characteristics of secondary school students in Owo, Ondo State, 2014

Sociodemographic Characteristics	Frequency	Percent
**Age group in years**		
<15	307	69.77%
15 and above	133	30.23%
**Sex**		
Female	212	48.18%
Male	228	51.82%
**Ethnicity**		
Yoruba	363	82.50%
Others	77	17.50%
**Religion**		
Christianity	367	83.41%
Islam	73	16.59%
**Class**		
Junior	220	50.00%
Senior	220	50.00%

Only 20(4.6%) said they have not heard of Ebola Virus Disease. Among those who have heard of EVD, level of hand washing with soap and water improved in 62.6%. Table 2 shows the socio-demographic factors associated with regular hand washing with soap and water.Regular hand washing with soap and water was reported among 119(56.94%) students in Junior Class and 144(68.25%)of those in senior class (p = 0.02).

**Table 2:** Socio demographic factors associated with regular hand washing with soap and water, Owo, Ondo State, 2014

Sociodemographic Characteristics	Regular hand washing with soap and water		Chi-square	p-value
	Yes	No		
**Age group in years**				
<15	172(59.52%)	117(40.48%)	3.81	0.05
15 and above	91(69.47%)	40(30.53%)		
**Sex**				
Female	128(64.32%)	71(35.68%)	0.47	0.49
Male	135(61.09%)	86(38.91%)		
**Ethnicity**				
Yoruba	216(61.89%)	133(38.11%)	0.47	0.49
Others	47(17.87%)	24(33.80%)		
**Religion**				
Christianity	127(35.80%)	228(64.2%)	2.53	0.11
Islam	30(46.2%)	35(53.8%)		
**Class**				
Junior	119(56.94%)	90(43.06%)	5.74	0.02
Senior	144(68.25%)	67(31.75%)		

Table 3 shows the source of information on Ebola Virus Disease and its association with regular hand washing with soap and water. Among those who heard about EVD through the television 205(72.44%) had regular hand washing with soap and water while only 58(42.34%) did not hear it through television (p < 0.001). Likewise 116(76.32%) who heard in Churches had regular hand washing with soap and water while only 147(54.85%) did among those who did not hear about it in the church(p < 0.001).

**Table 3:** Source of information on Ebola Virus Disease and its association with regular hand washing with soap and water, Owo, Ondo State, 2014

Source of information on Ebola	Regular hand washing with soap and water		Chi-square	p-value
	Yes	No		
**Social media**				
Yes	125(75.76%)	40(24.24%)	20.04	<0.001
No	138(54.12%)	117(45.88%)		
**Newspaper**				
Yes	174(70.45%)	73(29.55%)	15.69	<0.001
No	89(51.45%)	84(48.55%)		
**Radio**				
Yes	225(65.60%)	118(34.40%)	7.09	0.008
No	38(49.35%)	39(50.65%)		
**Family Members**				
Yes	104(75.36%)	34(26.64%)	14.26	<0.001
No	159(56.38%)	123(43.62%)		
**Flyers**				
Yes	14(70.00%)	6(30.00%)	0.49	0.48
No	249(62.25%)	151(37.75%)		
**Church**				
Yes	116(76.32%)	36(23.68%)	19.09	<0.001
No	147(54.85%)	121(45.15%)		
**Peers**				
Yes	45(77.59%)	13(22.41%)	6.44	0.01
No	218(60.22%)	144(39.78%)		
**Television**				
Yes	205(72.44%)	78(27.56%)	35.74	<0.001
No	58(42.34%)	79(57.66%)		

Table 4 shows the adjusted odds ratio of predictors of change in hand washing among secondary school students in Ondo State. After adjusting for age and class those who heard about EVD through the television were 2.2 times likely to have regular hand washing with soap and water (p = 0.02). Respondents who heard about EVD in Church were 2.4 times likely to have regular hand washing with soap and water (p = 0.01).

**Table 4:** Adjusted Odds Ratio of predictors of change in hand washing among secondary school students in Ondo State, 2014

Source of Information	Adjusted Odds ratio	95% CI		P-value
		Lower	Upper	
**Television**				
Yes	2.2	1.1	4.3	0.02
No	Ref			
**Church**				
Yes	2.4	1.2	4.7	0.01
No	Ref			

**Figure 1:**
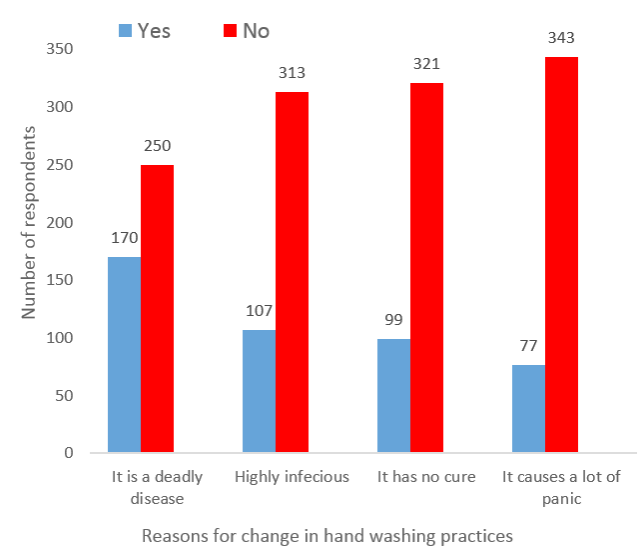
Reasons for change in hand washing practices among secondary school students in Ondo State, 2014

Figure 1 shows the reasons for change in hand washing practices. The main reason reported for change in hand washing practices among 170(40.5%) was because EVD causes death. Other reasons are shown in Figure 1.

## Discussion

Ebola virus genus has five subspecies belonging to the family of filoviridae and was first discovered in 1976 near the Ebola River in the now Democratic Republic of the Congo, the outbreak of the disease (Ebola hemorrhagic fever) caused by the virus then was sporadic. The natural reservoir is not yet established but studies have shown that the first patient became infected through contact with an infected animal and transmitted to other persons through direct contact with secretions and caring for ill persons [2].

The 2014 epidemic is enormous in history and was declared a global emergency by WHO in August, 2014 [2]. It has affected more than one country with the largest number of cases and case fatality rate in Liberia [2]. Though Nigeria, has been declared Ebola free by WHO in October 2014 [7] the early detection of the index case and combination of aggressive contact tracing efforts of all contacts with the index case helped to contain the outbreak [8] and intense campaign against the disease was done via various means such as the mass media, health education and school advocacy visits with emphasis on general public protection and control measures. Hand washing and the use of hand sanitizers were overtly emphasised. Hence the need to study the effect of some of the interventions on Nigerians’ level of hand washing with soap and water as a form of hygiene practices.

In this study, only 4.6% said they had not heard of Ebola Virus Disease. This proportion is low. However, with the urgency given to the dissemination of information about EVD in Nigeria within the first two months the first case was seen, all the respondents were expected to have heard.

Among secondary school students who have heard about EVD, level of hand washing with soap and water improved in more than half. School children often play important role in improving their behaviour and that of their family as well, because of this they have been seen as agents of change [9]. The importance of hand washing in viral diseases has been emphasised [10, 11]. Good hand hygiene is an important infection control measure for the prevention of transmission of gastrointestinal and respiratory infections [12].

Improvement in hand-washing was higher among those who watched EVD health messages on television and those who listened to it in the church. Several television campaign were aired during the EVD outbreak in Nigeria. A national emergency hand washing campaign was launched in Nigeria [13]. The use of the television yielded more results since the viewers had the opportunity to see hand washing been demonstrated. Television has been used in disseminating such messages in Ghana [14]. Hand washing was promoted by several churches in Nigeria during the EVD outbreak.

The main reason for change in hand washing practices was because EVD causes death. When some changes in behaviour are done out of fear, such behavioural change may not be sustained. It is therefore likely that the positive hygiene practices upheld during the outbreak of EVD in Nigeria are likely to be abandoned.

## Conclusions

The health education interventions during the outbreak of EVD in Nigeria led to increase in the proportion of people practising hand washing with soap and water. Watching health education messages on television and listening to it in church are the determinants of change in hand washing practices. Promotion of hand washing with soap and water needs to be sustained to prevent other diseases. Training of students on prevention of EVD was conducted in selected schools.
